# Glycosylation in the Tumor Microenvironment: Implications for Tumor Angiogenesis and Metastasis

**DOI:** 10.3390/cells8060544

**Published:** 2019-06-05

**Authors:** Kevin Brown Chandler, Catherine E. Costello, Nader Rahimi

**Affiliations:** 1 Center for Biomedical Mass Spectrometry, Department of Biochemistry, Boston University School of Medicine, Boston, MA 02118, USA; cecmsms@bu.edu; 2 Department of Pathology and Laboratory Medicine, Boston University School of Medicine, Boston, MA 02118, USA

**Keywords:** glycosylation, tumor microenvironment, angiogenesis, metastasis, *N*-glycosylation, *O*-glycosylation, glycosaminoglycans, endothelial, hypoxia, inflammation

## Abstract

Just as oncogene activation and tumor suppressor loss are hallmarks of tumor development, emerging evidence indicates that tumor microenvironment-mediated changes in glycosylation play a crucial functional role in tumor progression and metastasis. Hypoxia and inflammatory events regulate protein glycosylation in tumor cells and associated stromal cells in the tumor microenvironment, which facilitates tumor progression and also modulates a patient’s response to anti-cancer therapeutics. In this review, we highlight the impact of altered glycosylation on angiogenic signaling and endothelial cell adhesion, and the critical consequences of these changes in tumor behavior.

## 1. Introduction

A thin layer of endothelial cells lines the interior surfaces of blood and lymphatic vessels, releases signals that control vascular relaxation and contraction, secretes factors that regulate blood clotting, and plays an important role in immune function and platelet adhesion. The relationship between tumor cells and endothelial cells is complex. To sustain rapid cellular proliferation and a high metabolic rate, solid tumors develop a vascular network that fulfills tumors’ need for nutrients and oxygen and also aids in the removal of metabolic waste products [1]. In rapidly growing tumors, an angiogenic switch, often triggered by hypoxia-induced expression of vascular endothelial growth factor (VEGF) and other angiogenesis-inducing molecules, causes normally quiescent endothelial cells to proliferate and sprout [2,3,4,5,6]. In the tumor microenvironment (TME), dysregulation of angiogenic signals contributes to the development of hyper-permeable and highly heterogeneous blood vessels, and also aids in entry of tumor cells into (intravasation) and out of (extravasation) the blood stream via trans-endothelial migration [7]. Tumor-associated endothelial cells often exhibit decreased adhesion between neighboring cells and with the extracellular matrix, with profound consequences relevant to the development and treatment of cancer. Consequently, abnormally organized and leaky tumor blood vessels contribute to tumor angiogenesis, inflammatory cell infiltration, metastasis, and the development of resistance to chemotherapeutic agents in tumors of diverse origin [2,7,8,9,10,11,12,13].

Key instigators of angiogenic signaling, e.g., vascular endothelial growth factor receptor-2 (VEGFR-2), fibroblast growth factor receptor-1 (FGFR-1), Notch and Tie receptors, and critical endothelial adhesion molecules including vascular endothelial cadherin (VE-cadherin, also called cadherin-5), integrins, and immunoglobulin-like cell adhesion molecules (Ig-CAMs), are highly glycosylated. Global changes in glycosylation of these receptors and others could have wide-ranging consequences for their biological activity and interactions with other molecules [14]. The discovery that galectins, which bind β-galactoside sugars on glycoproteins, bind specific glycosylated forms of VEGFR-2, trigger receptor activation, and mediate resistance to anti-VEGF therapies used to treat cancer, represents a major shift in understanding and targeting VEGFR-2 signaling in cancer and other angiogenesis-associated diseases [15,16]. In addition, enzymatic removal or mutation of specific VEGFR-2 glycosylation sites amplifies ligand-dependent VEGFR-2 activation and signaling [17], indicating that angiogenic signaling of VEGFR-2 also is affected by changes in glycosylation independently of galectins.

**Figure 1:**
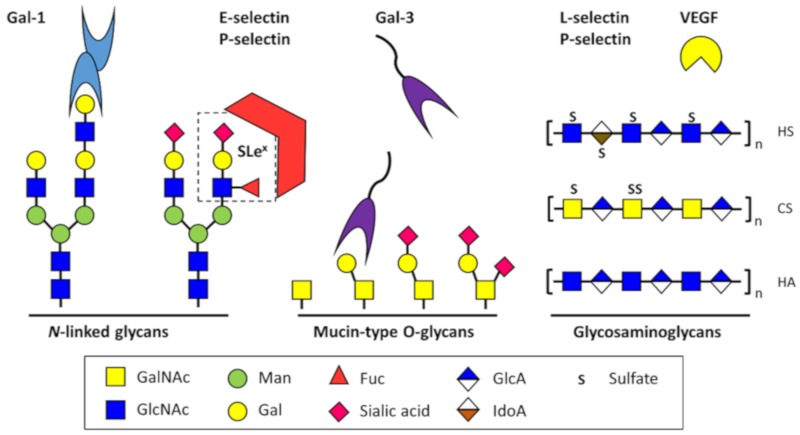
Major classes of glycans and glycosaminoglycans involved in endothelial cell signaling and adhesion. Representative glycan-binding lectins (Gal-1, Gal-3, E-selectin, P-selectin, and L-selectin), growth factors (such as vascular endothelial growth factor, VEGF), and glycoconjugates including *N*- and *O*-linked glycans, and glycosaminoglycans, are shown. Abbreviations: HS, heparan sulfate; CS, chondroitin sulfate; HA, hyaluronan; GalNAc, *N*-acetylgalactosamine; GlcNAc, *N*-acetylglucosamine; Man, mannose; Gal, galactose; Fuc, fucose; Sialic acid, *N*-acetylneuraminic acid; GlcA, glucuronic acid; IdoA, iduronic acid; S, sulfate.

Changes in glycosylation promoted by hypoxia, the shunting of glycolytic intermediates into glycan synthesis via the hexosamine biosynthesis pathway (HBP), and pro-inflammatory cytokines, have the potential to alter core physiologic characteristics of endothelial cells and contribute to dysregulation of endothelial signaling (Figure 1) [18,19,20,21]. There remains a critical need to understand how tumor microenvironment-induced changes in endothelial cell glycosylation alter angiogenic signaling, dysregulate adhesion, contribute to the formation of abnormal tumor vasculature, and promote tumor metastasis. The goal of this review is to highlight recent developments that have advanced our understanding of tumor microenvironment-directed changes in glycosylation that alter vascular endothelial cell signaling and adhesion and thereby contribute to tumorigenesis.

## 2. Glycoprotein and Glycosaminoglycan Synthesis and Recognition by Lectins

The luminal surface of endothelial cells contains an extensive network of membrane-bound glycoproteins and proteoglycans, called the endothelial glycocalyx. There is an increasing awareness that the endothelial glycocalyx plays a critical role in vascular physiology and pathology, especially with relation to tumor angiogenesis and interactions between endothelial cells and tumor cells that mediate trans-endothelial migration. Protein *N*- and *O*-glycosylation, as well as glycosaminoglycan (GAG) synthesis, involve multiple enzymatic steps that occur co- and/or post-translationally, are influenced by enzyme and substrate levels, and result in considerable structural diversity [22,23,24,25]. Tumor-associated endothelial cells are exposed to a hypoxic, hyper-glycolytic, and pro-inflammatory milieu [26,27,28]. Endothelial cell glycosylation is supremely sensitive to hypoxia and inflammation [29,30,31,32]. Tumor-associated endothelial cells adopt a hyper-glycolytic metabolic state [27,33,34]. The enzyme fructose-6-phosphate-amidotransferase (GFAT) converts fructose-6-phosphate into glucosamine-6-phosphate, and in so doing shunts glycolytic intermediates into the HBP, linking metabolism and glycosylation [35,36,37]. Glucosamine-6-phosphate is the common precursor to all amino sugars used in glycoprotein synthesis [38,39]. Ultimately, changes in endothelial cell glycosylation alter protein interactions and function at the plasma membrane [36,37,40,41,42,43].

Protein glycosylation changes dramatically in cancer, and has been studied extensively in tumor epithelial cells, where it regulates cellular adhesion, cell-matrix interactions, and signaling via receptor tyrosine kinases (RTKs) [40,44,45,46,47,48,49]. In fact, ST6Gal-I, responsible for the attachment of sialic acid to glycoproteins via 2,6-linkage, regulates transcription factors involved in stem cell maintenance [50]. However, until recently there has been little understanding of how changes in endothelial cell glycosylation in the tumor microenvironment influence endothelial barrier function, adhesion, cell-matrix interactions, and cell signaling.

### 2.1. N-Glycosylation

*N*-glycosylation occurs on asparagine (N) residues within the NXS/T motif, where any amino acid X except for proline (X ≠ P) follows asparagine, and serine or threonine (S/T) occupy the third position. *N*-glycosylation is a complex, multi-step co- and/or post-translational process that is initiated by the transfer of *N*-acetyl-glucosamine-1-phosphate (GlcNAc-1-P) to a dolichol-phosphate on the cytoplasmic face of the endoplasmic reticulum (ER) membrane by GlcNAc-1-phosphotransferase (encoded by the human *DPAGT1* gene, yeast *ALG7*) [23}. Notably, tunicamycin, an analog of uridine diphosphate-*N*-acetylglucosamine (UDP-HexNAc), inhibits this step and has been used widely to study *N*-glycosylation. After this initial step, an additional *N*-acetylglucosamine (GlcNAc) and five mannose (Man) residues are added sequentially. Then, the entire dolichol-linked glycan is flipped into the ER lumen, where four additional- Man residues and three glucose residues are added. This precursor, assembled from 14 monosaccharides, is then transferred by multi-subunit enzyme, oligosaccharyltransferase (OST), to an asparagine residue within the NXS/T motif. The nascent glycoprotein next undergoes interaction with chaperones to ensure quality control. Glycoproteins that ‘pass’ this quality control step proceed through multiple steps and are trimmed during protein folding to remove glucose. Further trimming and processing occurs in the ER and Golgi and produces a heterogeneous set of *N*-linked glycans.

### 2.2. Mucin-Type O-Glycosylation

Mucin-type *O*-glycans, also called *O*-GalNAc glycans, are initiated by the transfer of *N*-acetylgalactosamine (GalNAc) by polypeptide GalNAc-transferases (ppGalNAcTs) to specific Ser and Thr residues on *O*-glycosylated proteins. There are 20 human polypeptide GalNAc-transferase genes. This process occurs in the Golgi apparatus. *O*-glycosylated regions of proteins are frequently rich in serine, threonine and proline residues. *O*-glycans are commonly found on mucins, a class of glycoproteins that may each contain hundreds of such *O*-glycans, but other proteins can also be *O*-glycosylated, including membrane-associated glycoproteins such as P-selectin glycoprotein ligand 1 (PSGL-1). While all mucin-type *O*-glycans start with *O*-GalNAc there is considerable structural variability. There are four common *O*-glycan core structures, and additional rare core structures have also been elucidated [22].

### 2.3. O-GlcNAc

In contrast to the complex glycans on cell surface glycoproteins, *O*-linked β-*N*-acetylglucosamine (*O*-GlcNAc) modification of Ser and Thr residues occurs on intracellular proteins and is involved in signaling and the regulation of enzyme activity [51]. Two key enzymes, *O*-GlcNAc transferase (OGT) and *O*-GlcNAcase (OGA), catalyze the addition and removal of *O*-GlcNAc, respectively, from intracellular proteins. *O*-GlcNAc modification of endothelial nitric oxide synthase (eNOS) results in inactivation of the phosphorylated enzyme in the context of diabetes [52,53]. In addition, elevated flux through the HBP leads to increased protein modification by *O*-GlcNAc and impairs angiogenesis, potentially by inhibiting Akt signaling in endothelial cells [54]. Decreasing levels of OGT in prostate cancer cells diminished expression of VEGF and reduced endothelial tube formation in vitro, and regulation of this process involved FOXM1 [55].

### 2.4. Glycosaminoglycans

Glycosaminoglycans (GAGs) are long unbranched polysaccharides with repeating disaccharide units that are a major component of the extracellular matrix (ECM). They undergo sulfation at distinct positions and also undergo epimerization of uronic acid, resulting in the generation of a diverse set of molecules with distinct physical and biological properties. With the exception of hyaluronan, GAGs are covalently linked via serine residues to GAG-bearing proteins (proteoglycans) that reside on the cell surface and within the ECM. Six classes of GAGs exist, including chondroitin sulfate, dermatan sulfate, heparan sulfate, heparin, hyaluronan, and keratan sulfate. Hyaluronan (HA), a high-molecular-weight, non-sulfated glycosaminoglycan, is synthesized at the cell surface and is subsequently incorporated into the extracellular matrix [24,45].

### 2.5. Glycan-Binding Proteins

Lectins are a class of glycan-binding proteins that recognize carbohydrate substructures within larger branched carbohydrates. Lectins are notable for their low-affinity interactions, which mediate “rolling” in of leukocytes and cancer cells when they interact with glycans on the endothelial cell surface. In this review, we will discuss two major classes of lectins, which are categorized by the substructures they bind. The first, galectins, recognize glycans with exposed galactose residues [56]. The second, selectins, are a family of calcium-dependent cell adhesion molecules that recognize sialylated, fucosylated carbohydrate ligands with low affinity [57]. Selectins are upregulated in inflammatory conditions to recruit platelets and leukocytes to sites of injury or infection, but may also be co-opted in the context of cancer to facilitate tumor cell adhesion to endothelial cells. In addition to lectins, a broad array of molecules, including many growth factors, have the ability to bind with carbohydrate moieties on glycoproteins and glycosaminoglycans, and in so doing they mediate cell–cell and cell–matrix interactions.

The modification of membrane glycoproteins by *N*- and *O*-glycans, cytoplasmic *O*-glycosylation, the production and deposition of glycosaminoglycans, and the recognition of motifs on glycoconjugates by lectins, have been characterized extensively in the epithelial context. Next, we will discuss specific glycoproteins that are involved in endothelial cell adhesion, and how carbohydrate modifications may impact the function of these molecules.

## 3. Endothelial Cell Adhesion Molecules

Much of what is known about the impact of altered glycosylation on cell–cell and cell–matrix adhesion is derived from studies of aberrant glycosylation in tumor cells [58,59,60]. For example, increased β-1,6 branching and increased sialylation on *N*-linked glycans that occurs during tumorigenesis lessens cell–cell adhesion [58,61]. In contrast, knowledge of the impact of altered glycosylation on endothelial adhesion molecules is primarily based on the interaction of endothelial cell adhesion molecules with immune cells in the context acute inflammatory conditions. Glycans on the surface of leukocytes, and to a lesser extent, glycans on the surface of endothelial cells, play a crucial role in leukocyte recruitment. Glycosyltransferases, including α1,3 fucosyltransferases, α2,3 sialyltransferases, core 2 *N*-acetylglucosaminlytransferases, β1,4 galactosyltransferases, and polypeptide *N*-acetylgalactosaminyltransferases are involved in the synthesis of selectin ligands that mediate leukocyte rolling by binding to selectins [62]. Major glycoconjugates and lectins involved in endothelial cell adhesion and signaling are shown in Figure 1.

### 3.1. ICAM-1

Intercellular adhesion molecule 1 (ICAM-1/CD54) is involved in trans-endothelial migration of leukocytes and serves as a ligand for integrins on leukocytes. Scott et al. (2013) showed that activated endothelial cells expressed two forms of ICAM-1, the more abundant of which displayed *N*-glycans modified with α2,6-linked sialic acids, while the less abundant form displayed primarily high-mannose type glycans. Inhibition of α-mannosidase to force expression of high-mannose *N*-glycans led to increased monocyte rolling and adhesion, as compared with ICAM-1 displaying more processed *N*-glycans, suggesting that the high-mannose glycans could serve as leukocyte ligands. However, in cells with ICAM-1 displaying high-mannose glycans, interactions with the actin cytoskeleton were lost, suggesting that the glycosylation status and adhesion properties of ICAM-1 are modulated by inflammation [63].

### 3.2. Endothelial Selectins: E-Selectin (ELAM) and P-Selectin

E-selectin is an endothelial-specific lectin that recognizes glycans containing the sialyl-Lewis x substructure (SLe^x^; NeuAc α2,3Gal β1,4(Fuc α1,3)-GlcNAc, its expression is activated by cytokines, and it is involved in recruitment of neutrophils to sites of inflammation [64,65]. Aberrant expression of glycans bearing the SLe^x^ motif in multiple types of cancer, including colon cancer [66,67] and prostate cancer [68,69,70], has been implicated in facilitating tumor cell adhesion to the endothelial cells, and facilitating tumor cell metastasis via interaction with selectins [71,72]. P-selectin is expressed in both platelets and activated endothelial cells. In endothelial cells, P-selectin is stored in Weibel–Palade bodies and is rapidly released and translocated to the cell surface in response to inflammation. P-selectin glycoprotein ligand-1 (PGSL-1/CD162) is a ligand of P-selectin that is expressed on leukocytes and contains mucin-type *O*-glycans. Interestingly, P-selectin deficient mice show a decreased rate of tumor growth and decreased metastasis compared to wild-type mice [73]. This can be explained in part by the fact that tumors frequently express glycosylated ligands with sialyl-Lewis x structures, bind platelets and leukocytes via P-selectin, and use these interactions to initiate contact with endothelial cells at distant sites and extravasate [74]. Some tumor cells express P-selectin and initiate this process in a platelet-independent manner [75].

### 3.3. VCAM-1

Vascular cell adhesion molecule 1 (VCAM-1/CD106) is an endothelial glycoprotein, its expression is upregulated in response to TNF-α, IL-1 and IL-4, and it is involved in leukocyte adhesion to endothelial cells, an interaction mediated by VCAM-1 binding to α4 integrins (i.e., α4β1 and α4β7) on leukocytes. In response to IL-1 and IL-4, α2,6-sialyltransferase (ST6Gal-I) expression is enhanced. A decrease in α2,6-linked sialic acids increased VCAM-1-dependent adhesion, while α2,3-linked sialic acids did not impact adhesion [76].

### 3.4. PECAM (CD31)

Platelet endothelial cell adhesion molecule (PECAM) is involved in cell adhesion, mechanical stress sensing, angiogenic signaling, and also has an anti-apoptotic role [77]. It is a major component of intercellular junctions in endothelial cells. In addition, it has been shown to have lectin-like properties and recognize α2,6-sialic acid, and this property is involved in regulation of hemophilic interactions [78,79]. PECAM glycans bearing α2,6-sialic acid are essential for endothelial tube formation, and removal of these sialic acid residues disrupts endothelial tube formation [80,81]. Several *N*-glycans are located at the hemophilic binding interface [82], suggesting that α2,6-sialylated glycans modulate homophilic PECAM-dependent interactions. A decrease in α2,6-sialylation reduces the levels of PECAM at the cell surface and increases its role in apoptosis, and may regulate interactions between PECAM, VEGFR2, and integrin-β3 [77,83]. Therefore, α2,6-sialylated glycans appear to be critical for endothelial cell survival, as they stabilize membrane proteins, leading to their retention at the cell surface and thereby impact pro-angiogenic signaling.

### 3.5. IGPR-1

The Ig-containing and proline-rich receptor-1(IGPR-1) is a newly identified Ig-CAM that is uniquely expressed in human and other higher mammalians, but not in rodents [84]. IGPR-1 is expressed in endothelial cells and regulates endothelial barrier function and angiogenesis [85]. More importantly, IGPR-1 expression is elevated in various tumors including, colon cancer [86]. Although it is heavily glycosylated [84], the role of glycosylation in IGPR-1 function has not been studied.

### 3.6. VE-Cadherin

Vascular endothelial cadherin (VE-cadherin/CD144) is an endothelial-specific adhesion molecule that is an essential player in the formation of cell–cell endothelial adherens junctions and controls vascular permeability. Analyses of VE-cadherin *N*-glycans indicate that it bears predominantly sialylated biantennary and hybrid-type glycans, and it may also be *O*-manosylated [87,88]. Sialic acid-bearing glycans on VE-cadherin are likely important for maintenance of endothelial cell adherens junctions [89].

### 3.7. Endomucin

Endomucin is a highly sialylated, type I *O*-glycosylated protein that is endothelial specific. Endomucin is involved in angiogenesis [90], and recent evidence suggests that the α1,3-fucosyltransferase FUT7, upregulated by IL-1β, induced monocyte-endothelial adhesion via fucosylation of endomucin [91].

As we have noted here, several of the glycoproteins and glycan-binding proteins discussed above, including ICAM-1, E-Selectin, P-Selectin, VCAM-1, and PECAM, are known to initiate specific adhesive interactions only when modified (or binding to) specific glycan substructures. As a result, changes in glycosylation alter the functions of these proteins. Below, we will discuss factors that influence endothelial glycosylation and in so doing alter endothelial cell adhesion.

## 4. Factors that Influence Endothelial Glycosylation

Endothelial glycosylation is evolutionarily conserved in both developmental and inflammatory processes. Yano et al. (2007) examined the endothelium of hagfish to understand evolutionarily-conserved features of the endothelium using lectins LCA (*Lens culinaris* agglutinin) and HP (*Helix pomatia*) that bind carbohydrate structures containing α-linked mannose and α-*N*-acetylgalactosamine respectively, to characterize differences in glycosylation between endothelial cells in different vascular beds. Their analyses revealed that vascular bed-associated differences in glycosylation facilitated histamine-induced adhesion of leukocytes in capillaries and post-capillary venules but not in the aortic endothelium or arterioles, suggesting a link between inflammation and altered glycosylation [92]. Using similar methods, Jilani et al. (2003) demonstrated that lectin affinities differed between the vasculature of chicken embryos at early and late stages of development, suggesting that endothelial glycosylation plays a role in embryonic development [93]. These patterns are likely relevant to human to the biology of human cells as well.

Inflammatory cytokines TNF-alpha and interleukin-1, and bacterial lipopolysaccharide increase expression of ST6Gal-I and also increase the binding of lectins with affinity for sialic acid to the endothelium. E-selectin, ICAM-1, and VCAM-1 were reported as glycoprotein substrates for ST6Gal-I [94]. DW Scott et al. demonstrated that inflammatory stimuli including TNF-α, LPS, and IL-1β induce changes in expression of specific endothelial glycoproteins involved in monocyte adhesion including ICAM-1 and VCAM-1, as well as expression of enzymes involved in *N*-glycan processing including α-mannosidase, which catalyzed the removal of two mannose residues from GlcNAcMan5GlcNAc2, the committed step in the synthesis of complex *N*-linked glycans [95]. These investigators showed that endothelial responses to inflammatory stimuli vary between vascular beds [96].

Within the tumor microenvironment, inflammatory stimuli, hypoxia, and tumor-secreted signaling factors alter expression of endothelial cell surface carbohydrates by impacting the underlying expression of enzymes involved in carbohydrate synthesis [16,95,96,97]. Table 1 shows the reported impact of various cytokines and hypoxia on endothelial glycosylation. Pro-inflammatory signals including IFN-γ and IL-17 increase the expression of α2,6-linked sialic acid-containing carbohydrate epitopes on the endothelial cell surface glycoproteins. In contrast, immunosuppressive cytokines IL-10 and TGF-β1 reduce α2,6-linked sialic acid-containing carbohydrate epitopes on *N*-linked glycans [16]. In addition, tumor necrosis factor-α (TNF-α) and interleukin-1β (IL-1β) alter endothelial surface *N*-glycosylation and this correlates with increased monocyte adhesion [76,95]. Critically, immune-mediated mechanisms that alter glycosylation and the expression of glycan-binding proteins have been shown to lead to acquired resistance to anti-angiogenic therapies via changes in the interaction with glycan-binding proteins [98].

The impact of hypoxia on endothelial cells in the tumor microenvironment has been extensively studied [99,100,101]. Hypoxia-inducible factor (HIF-1) is a heterodimeric transcription factor composed of subunits HIF-1β/aryl hydrocarbon receptor nuclear translocator (ARNT) and either HIF-1α or HIF-2α. Under normoxic conditions, prolyl-hydroxylase (PHD) enzymes including PHD2 hydroxylate HIFα, leading to HIF-1 inactivation, followed by its ubiquitination by the von Hippel–Lindau tumor suppressor (pVHL), an E3 ubiquitin ligase, and subsequent degradation [102,103,104]. However, under hypoxic conditions such as those in the tumor microenvironment, PHD2, unable to bind oxygen, no longer hydroxylates HIFα, and this results in its accumulation [105]. HIF-1α and HIF-2α regulate different and, in some cases, opposing, sets of genes [106,107]. While there is some evidence that HIF-1 signaling alters glycosylation, the extent of its influence on endothelial glycosylation, the potential differential roles of HIF-1α and HIF-2α, and the physiological impact of the resulting changes in glycosylation are unclear. In addition to hypoxia, the role of metabolism in endothelial cell glycosylation is an intriguing, though unexplored, subject. It has been reported that the glycolytic activator PFKFB3 regulates endothelial cell rearrangement during vessel sprouting, in part by reducing intercellular adhesion [108]. The role of glycosylation in reducing intercellular adhesion should be further investigated in this context.

Abnormal endothelial cell glycosylation and increased expression of lectins, which bind glycan epitopes, aid the development of resistance to anti-angiogenic cancer therapeutics [14,109]. Further exploration should be done to understand the impact of these changes in both acute and chronic inflammation. Hypoxia, a common feature of the tumor microenvironment, also appears to alter endothelial cell glycosylation, leading to the production of glycoproteins bearing carbohydrate structures with less α2,6-linked sialic acid, greater branching of β1,6 *N*-glycan structures, and elongation with poly-LacNAc residues [16]. Culturing endothelial cells in a tumor conditioned medium from colon carcinoma cell line HT29 induced increased β1,6-GlcNAc branching of endothelial cell glycans, suggesting that factors secreted by tumor cells also influence glycosylation in their environment [32]. Inflammatory cues, hypoxia, and tumor-secreted factors, by triggering changes in endothelial surface carbohydrate structures, may alter angiogenic signaling by modifying the properties of endothelial glycoproteins that are key mediators of signaling and adhesion. To improve our understanding of the role these changes play in tumor progression, metastasis, and treatment, further study is required in animal models and human tissue.

## 5. Glycosylation and VEGFR2 Pro-Angiogenic Signaling

Vascular endothelial growth factors (VEGFs), first identified based on their role in vascular permeability, bind to extracellular matrix proteoglycans (specifically, heparan sulfate proteoglycans, HSPGs), resulting in their sequestration and controlled release from the extracellular matrix in cases of tissue damage or remodeling by matrix-metalloproteinases. Upon release, they are available to promote angiogenesis to repair tissue, although this process is dysregulated in the tumor microenvironment. Additional factors, including fibroblast growth factors, and angiogenic inhibitors such as thrombospondin and platelet factor 4, also interact with and are in some instances stabilized by HSPGs [110]. Using proximity ligation assays in primary brain endothelial cells, Xu et al. (2011) demonstrated that heparan sulfate and VEGFR2 interact directly, and that the number of heparan sulfate-VEGFR2 complexes increased in response to stimulation with VEGF_165_ and VEGF_121_ [111]. HSPGs also bind gremlin (Drm), and alter its activation of VEGFR2 [112].

Most endothelial surface proteins bear *N*- and/or *O*-linked glycans. Multiple adhesion molecules bind glycoconjugates expressed on the surfaces of endothelial cells [113]. The cell-surface receptor tyrosine kinase VEGFR2 is involved in pro-angiogenic signaling in endothelial cells and plays a critical role in tumor angiogenesis. The extracellular domain of VEGFR2 is highly modified by *N*-linked glycans [114], and glycans, especially α2,6-linked *N*-glycans at site N247 on Ig-like domain 3 near the ligand binding pocket, influence ligand-dependent signaling [17]. Immune-mediated mechanisms that alter glycosylation and influence endothelial cell signaling are implicated in acquired resistance to anti-angiogenic therapies, highlighting the convergence of immunosuppressive and pro-angiogenic signaling in the tumor microenvironment. Chiodelli et al. (2017) also found that VEGFR2-associated NeuAc plays an important role in modulating VEGF/VEGFR2 interaction, pro-angiogenic activation of endothelial cells and neovascularization [14].

**Figure 2:**
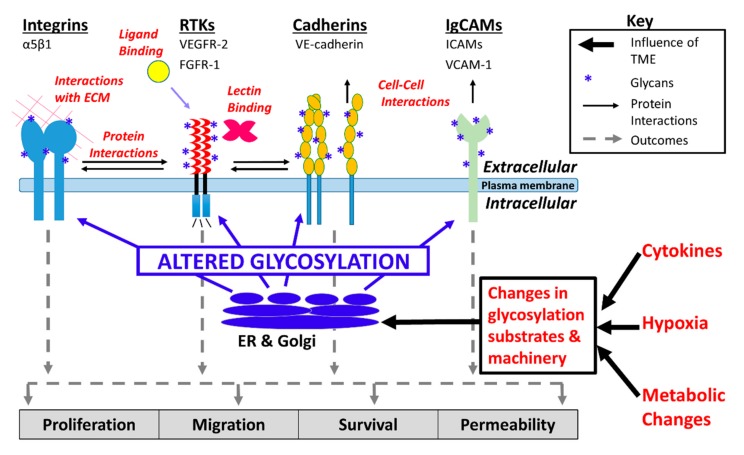
Tumor microenvironment-mediated changes in endothelial cell glycosylation. Endothelial glycoproteins are shown, including integrins, receptor tyrosine kinases (RTKs), VE-cadherin, and Ig-like cell adhesion molecules (IgCAMs). Glycans synthesized in the endoplasmic reticulum (ER) and Golgi have the potential to alter signaling and adhesion.

Galectin-3 (Gal-3) is able to induce angiogenesis in a glycan-dependent manner by binding to glycoproteins on the surface of endothelial cells [15]. VEGFR-2 *N*-glycans are involved in retention of the receptor at the endothelial cell surface via interaction with Gal-3 [115]. Rabinovich et al. studied anti-VEGF refractory tumors and found that glycans on the endothelial surface glycoproteins, including VEGFR2, were remodeled to selectively bind galectin-1 (Gal-1) expressed by the tumor cells. Endothelial cells displayed high levels of β1,6-GlcNAc-branched *N*-glycans and low levels of α2,6-linked sialic acid in anti-VEGF refractory tumors compared to tumors that were sensitive to anti-VEGF treatment. Binding of Gal-1 to VEGFR2 resulted in VEGF-*independent* activation of the receptor [16]. The group also found that hypoxia upregulates expression of galectin-1 (Gal-1) via HIF-1-dependent and -independent mechanisms. In Kaposi’s sarcoma, activation of the transcription factor nuclear factor κB (NF-κB) by reactive oxygen species resulted in higher levels of Gal-1 expression that promoted angiogenesis and tumorigenesis [116]. In another study by the same group, HIF-1α was found to increase Gal-1 expression in colorectal cancer (CRC) cells, and the group identified two hypoxia-responsive elements upstream to the transcriptional start site of the Gal-1 gene that are essential for HIF-1-mediated galectin-1 expression [16]. Tumor microenvironment-dependent changes in endothelial cell glycosylation are summarized in Figure 2.

## 6. Glycosaminoglycans in Tumor Angiogenesis and Metastasis

Within the ECM, GAGS play a role in regulating migration of endothelial cells, providing a scaffold that guides endothelial cell tube formation, and stabilizes neovasculature. An excellent review by Oliveira-Ferrer, et al. describes the varied roles of GAGs in metastasis [117]. Here, we will primarily discuss the role of GAGs as they relate to endothelial cell function (or dysfunction) in cancer.

### 6.1. Heparan Sulfate Proteoglycans (HSPGs)

HSPGs are a well-studied group of proteins that bear long heparan sulfate chains consisting of 50–200 glucuronic acid disaccharide repeats with variable patterns of sulfation, and reside both on the endothelial cell surface and within the extracellular matrix. HSPG modifications including sulfation create binding sites for various ligands, including adhesive proteins, chemokines, growth factors and growth factor-binding proteins, proteases and protease inhibitors, and morphogens [118,119,120,121,122]. Critically, these interactions are sensitive to the position and linkage of sulfate modifications. Transmembrane HSPGs including syndecans, glycpicans, and perlecan reside on the cell surface and are involved in extracellular matrix assembly and maintenance. Both VEGFR2 and VEGF (including VEGF_165_ but not VEGF_121_) interact with heparan sulfate, and ligand-stimulation has been reported to increase heparan sulfate-VEGFR2 complex formation and vascular permeability [111]. VEGF HS-binding domains encoded by exons 6 and 7 are responsible for the interaction of VEGF ligands with HS, and result in the sequestration of VEGF in the extracellular matrix that may subsequently be released by proteases and heparanase during ECM degradation by proteases associated with angiogenesis [123,124,125]. The ability of VEGF_165_ to bind HS is partially controlled by its interaction with endothelial transglutaminase-2 [126].

Additional growth factors, including PDGF-B, contain HS-interacting domains [127,128]. TGF-β isoforms also bind HS, and HS plays a role in gradient formation of cytokines [129,130]. By regulating heparan sulfate modifications on endothelial cells, heparan sulfatases affect tumor angiogenesis in a number of contexts, including ovarian and breast cancer. Downregulation of endosulfatases responsible for removal of 6-*O* sulfate from HS in response to hypoxia, as well as downregulation in tumor cells, results in the presence of more highly sulfated forms of HS, thus increasing growth factor binding and downstream signaling [131].

### 6.2. Chondroitin Sulfate (CS)

Chondroitin sulfate (CS), composed of repeating units of the disaccharide GalNAc-GlcA, is also variably-sulfated in a tissue-specific manner by carbohydrate sulfotransferases. Expression of specific sulfated forms of CS on the surface of tumor cells facilitates their interaction with platelets and endothelial cells by creating ligands that bind P-selectin, e.g., in breast cancer [132]. Moreover, the sulfation pattern of CS on versican appears to be critical for interaction with L-selectin, P-selectin, and CD44, molecules involved in endothelial cell adhesion and/or tumor angiogenesis [133]. However, the full role of such modifications in tumor angiogenesis remains to be determined. 

### 6.3. Hyaluronan (HA)

Hyaluronan (HA) is a negatively charged, nonsulfated GAG. Unlike other GAGs, hyaluronan (HA) is not covalently linked to a core protein. Rather, it is deposited in the extracellular matrix, where it may interact with ECM proteins and other GAGs. In healthy tissue, the coordinated expression and activity of HA synthases and hyaluronidases maintain a homeostasis. In tumors, higher expression of low-molecular weight HA is often present and is associated with inflammatory conditions [134], and contributes to tumor angiogenesis by impairing cellular adhesion [135,136]. HA also seems to play a role in tumor-associated macrophage trafficking to tumor stroma [137].

## 7. Endothelial Glycosylation Regulates Tumor Cell Trans-Endothelial Migration

The binding to glycosylated epitopes on tumors by selectins (E-selectin, P-selectin) and galectins expressed on endothelial cells, and of tumor-expressed lectins to endothelial glycans, mediates a process of rolling followed by stable heterotypic adhesion. This process mirrors the process through which platelets and leukocytes interact with the endothelium. The glycan-binding proteins on endothelial cells recognize glycan substructures on platelets, leukocytes, and circulating tumor cells. Conversely, L-selectin expressed on leukocytes (specifically, T cells) also recognizes glycan structures on endothelial cells, allowing leukocytes to attach to specific endothelial beds, based purely on the glycans expressed on the endothelial surface [138]. Sulfated glycans also play a role in this process in lymphatic endothelium. There is evidence that these interactions are regulated by the spatial and temporal expression of glycosyltransferases and sulfotransferases in endothelial cells in a bed-specific manner, and by inflammatory signals.

Galectin-3 (Gal-3) expressed on endothelial cells is a major actor in tumor metastasis. Gal-3 is the only human lectin of the ‘chimera’ galectin subtype. It can exist as a monomer, or form multivalent complexes of up to five Gal-3 molecules via its non-lectin domain, allowing it to facilitate the interaction of multiple glycoproteins. By binding T antigen on MUC-1, Gal-3 promotes adhesion of tumor cells to the endothelium in breast and prostate cancer [139,140,141]. Circulating Gal-3 can also increase tumor cell adhesion to and migration across the endothelium by interacting with MUC1 on tumor cells, leading to exposure of additional glycosylated ligands including CD44 that bind E-selectin on endothelial cells [142]. Under flow conditions, highly metastatic MDA-MB-435 human breast carcinoma cells that express high levels of T antigen and Gal-3 showed increased adhesion to endothelial cells compared to similar non-metastatic cells [143].

**Figure 3:**
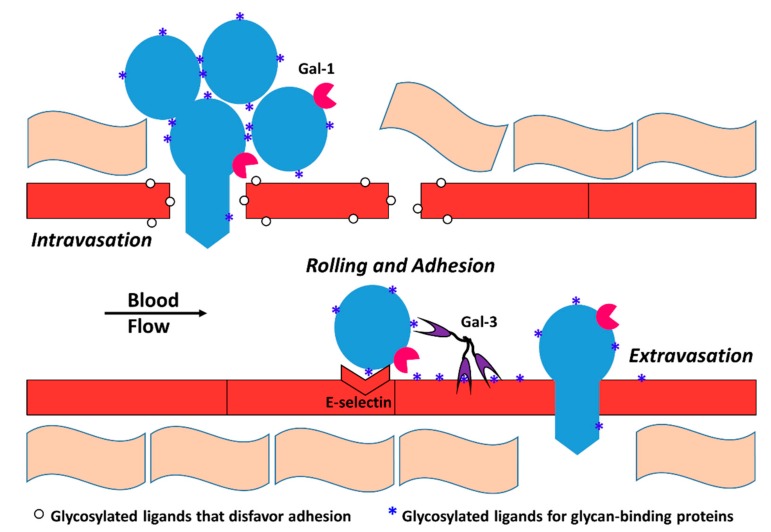
Glycan-mediated intravasation, rolling, and extravasation. Glycan modifications on endothelial cells (red) alter endothelial adhesion and may contribute to endothelial permeability and tumor cells (blue) intravasation. Circulating lectins such as Gal-3 and endothelial lectins including E-selectin initiate rolling, adhesion, and extravasation of tumor cells to the endothelium, frequently at distant sites. Integrins also assist in this process.

Glycan-mediated intravasation, rolling, and extravasation of tumor cells contribute to tumor metastasis (Figure 3). For example, in colon and prostate cancers, glycans with the SLe^x^ motif (a tetra-saccharide containing both sialic acid and α1,3 linked fucose) are involved in tumor metastasis [66,67]. Forced reduction in the expression of α1,3 fucosyltransferases reduced incidence of prostate cancer in mice [68,69,70]. As previously noted, this can be explained by tumor cell adhesion to the endothelial cells via interaction with selectins [71,72]. In patients with multiple myeloma, high expression of ST3Gal6, which catalyzes the 2,3-linked attachment of sialic acid residues to glycoproteins, correlates with lower overall survival. Knockdown of ST3GAL6 in multiple myeloma cells diminished the cells’ ability to undergo trans-endothelial migration and reduced ability to roll on P-selectin in vitro [144].

## 8. Toward Therapeutic Strategies that Target Endothelial Glycosylation

Several anti-cancer therapeutic strategies that target tumor vasculature have been proposed, and include (a) the inhibition of tumor angiogenesis and (b) treatments that promote blood vessel normalization to enhance delivery of chemotherapeutic agents and reduce metastasis [2,145,146,147,148]. In clinical trials, anti-angiogenic therapies have shown promise in patients with colorectal, lung, breast, and other cancers, but resistance to these therapies often develops rapidly [98,145,149,150,151]. Additional drug targets that aid in vascular normalization are being investigated [146]. There remain gaps in our understanding of tumor-associated endothelial cell pathobiology, including how tumor microenvironment-induced changes in the glycosylation of endothelial adhesion and signaling molecules contribute to altered angiogenesis. Addressing this gap in knowledge could lead to the design and delivery of pharmacological agents that aid in normalizing blood vessels, prevent metastasis and increase responsiveness to targeted chemotherapeutics.

A number of approaches that target protein glycosylation attempt to address this gap. Therapeutic targeting of glycan-mediated processes has been explored, including the use of glycomimetics [152]. Partial inhibition of OST, the enzyme involved in the initiation of *N*-linked glycosylation, is an approach pioneered by Contessa et al. [153,154]. Among the molecular targets of this strategy are receptor tyrosine kinases such as EGFR, which are highly *N*-glycosylated. The approach is currently being tested in a number of pre-clinical models [155]. While it has not been tested in the context of angiogenesis, it is notable that VEGFR2 and additional RTKs involved in pro-angiogenic signaling are highly *N*-glycosylated, and therefore might also be susceptible to targeting by this drug, potentially in combination with other approaches. Another breakthrough involves the development of fucosyltransferases inhibitor 2-fluorofucose (2-FF) by Okeley et al. (2013) [156]. Many selectin ligands are fucosylated, and administration of 2-FF could potentially block these interactions and attenuate trans-endothelial migration of tumor cells. In pre-clinical models, 2-FF inhibited leukocyte-endothelium interactions [157], inhibits liver cancer HepG2 proliferation, migration, and tumor formation [158], and reduced fucosylated E-selectin ligand expression in human invasive ductal carcinoma [159]. Multiple fucosyltransferases in humans catalyze the attachment of fucose via specific linkages to glycans. It is likely that the development of fucosyltransferase-specific inhibitors will ultimately be the most successful strategy, as this will enable targeting of specific fucose linkages involved in metastasis while minimizing off-target effects. Thioglycosides are a class of compounds that are currently being tested as glycosylated decoys to reduce selectin-dependent leukocyte adhesion [160]. It remains to be seen whether a similar approach might be applied in the context of cancer treatment. Additionally, targeting selectin-mediated cell adhesion to endothelial cells may represent an opportunity to control tumor immunity [161]. As discussed previously, heparanase is elevated in multiple types of cancer and promotes tumor invasion, angiogenesis, and metastasis. Heparanase inhibitors that prevent the release of heparan sulfate side chains have been tested in pre-clinical and clinical settings, and reduce tumor metastasis by maintaining ECM integrity and partially restoring vascular function [162,163,164,165].

## 9. Conclusions

Tumor-associated endothelial cells are significantly influenced by signals from nearby tumor cells, stromal cells and infiltrating immune cells. Glycans on endothelial adhesion molecules including ICAM-1, VCAM-1, and PECAM, and glycan-binding proteins (lectins) expressed on the surfaces of endothelial, immune, and cancer cells, alter the adhesive properties of endothelial cells and facilitate (or disfavor) immune and tumor cell infiltration. In addition, altered endothelial cell glycosylation in the tumor microenvironment has been shown to impact VEGFR2-mediated angiogenic signaling. Further investigation will be needed to understand how changes in tumor-associated endothelial cell glycosylation machinery, with cues from the tumor microenvironment, dysregulate endothelial cell signaling and adhesion, and contribute to the formation of abnormal and leaky tumor blood vessels. Since glycosylation is not template based, different sites within the same protein may be occupied by different glycan structures, and a single protein may have many glycoforms with different biological functions. Major barriers to progress in this field have included (a) the technical challenge of analyzing glycan heterogeneity, (b) the low abundance of plasma membrane receptors and adhesion molecules, and (c) the complexity of linking non-template-based protein glycosylation status to biological function. Despite these challenges, significant progress has been made towards elucidating the roles of normal and aberrant glycosylation in endothelial processes, and we further expect that advances will be made in these areas in the years ahead. We predict that recent advances in mass spectrometry-based methods for the characterization of glycoconjugates, in combination with gene expression analyses in model systems and tissue, CRISPR (clustered regularly interspaced short palindromic repeats)-Cas9 gene editing, and the application of fluorophore-conjugated lectins for live cell and tissue imaging, among others, will enable the establishment of a clear relationship between changes in glycan structures on the cell surface and altered endothelial function in tumor-associated endothelial cells. The knowledge gained in this exciting and emerging field of biology can lead to development of a new class of therapeutics to combat cancer and other diseases.

## Figures and Tables

**Table 1:** Factors that influence endothelial cell glycosylation. Pro- and anti-inflammatory actors that have been reported to impact endothelial cell glycosylation are shown.

Factor	Inflam.	Source(s)	Impact on Endothelial Glycosylation
TNF-α	Pro	macrophages, CD4+ lympho-cytes, NK cells, neutrophils	↑ ST6Gal-I and α-mannosidase expression [94,95]
IL-1*	Pro	macrophages, monocytes, fibroblasts, and dendritic cells	↑ ST6Gal-I expression [94]
IL-1β	Pro	macrophages, dendritic cells	↑ α-mannosidase expression [95]
IFN-γ	Pro	NK, NKT cells, and CD4+ T_h_1 and CD8+ CTL effector T cells	↑ α2,6-linked sialic acids [16] (presumably via ↑ ST6Gal-I expr.)
IL-17	Pro	T_h_ (CD4+) cells	↑ α2,6-linked sialic acids [16] (presumably via ↑ ST6Gal-I expr.)
IL-10	Anti	T_h_2, mast cells, CD4+ CD25+ Foxp3+ T_reg_	↓ α2,6-linked sialic acids [16] (presumably via ↓ ST6Gal-I expr.)
TGF-β1	Anti	Platelets, most leukocytes	↓ α2,6-linked sialic acids [16] (presumably via ↓ ST6Gal-I expr.)
Hypoxia	N/A	N/A	↓ α2,6-linked sialic acids, ↑ β1,6 branching, elongation of poly-LacNAc chains [16]
